# Polymer Gel Substrate: Synthesis and Application in the Intensive Light Artificial Culture of Agricultural Plants

**DOI:** 10.3390/gels9120937

**Published:** 2023-11-29

**Authors:** Gayane G. Panova, Elena L. Krasnopeeva, Svetlana G. Laishevkina, Tatiana E. Kuleshova, Olga R. Udalova, Yuriy V. Khomyakov, Galina V. Mirskaya, Vitaly E. Vertebny, Anna S. Zhuravleva, Natalia N. Shevchenko, Alexander V. Yakimansky

**Affiliations:** 1 Agrophysical Research Institute (AFI), 195220 Saint-Petersburg, Russia; www.piter.ru@bk.ru (T.E.K.); udal59@inbox.ru (O.R.U.); himlabafi@yandex.ru (Y.V.K.); galinanm@gmail.com (G.V.M.); verteb22@mail.ru (V.E.V.); zhuravlan@gmail.com (A.S.Z.); 2 Institute of Macromolecular Compounds, Russian Academy of Sciences (IMC RAS), 199004 Saint-Petersburg, Russia; opeeva@gmail.com (E.L.K.); s.laishevkina@gmail.com (S.G.L.); natali.shevchenko29@gmail.com (N.N.S.); yakimansky@yahoo.com (A.V.Y.)

**Keywords:** gel substrate, vegetable crops, photosynthetic apparatus, antioxidant systems, pigments, intensity of lipid peroxidation, peroxidase, catalase, growth, production, quality, safety, element content

## Abstract

This work is devoted to the description of the synthesis of hydrogels in the process of cryotropic gel formation based on copolymerization of synthesized potassium 3-sulfopropyl methacrylate and 2-hydroxyethyl methacrylate (SPMA-co-HEMA) and assessing the potential possibility of their use as substrates for growing plants in intensive light culture in a greenhouse. Gel substrates based on the SPMA-co-HEMA were created in two compositions, differing from each other in the presence of macro- and microelements, and their effects were studied on the plants’ physiological state (content of chlorophylls a and b, activity of catalase and peroxidase enzymes, intensity of lipid peroxidation, elemental compositions) at the vegetative period of their development and on the plants’ growth, productivity and quality of plant production at the final stages of development. Experiments were carried out under controlled microclimate conditions. Modern and standard generally accepted methods of gels were employed (ATR-FTIR and ^13^C NMR spectral studies, scanning electron microscopy, measurement of specific surface area and pore volume), as well as the methods of the physiological and chemical analysis of plants. The study demonstrated the swelling ability of the created gel substrates. Hydrogels’ structure, their specific surface area, porosity, and pore volume were investigated. Using the example of representatives of leaf, fruit and root vegetable crops, the high biological activity of gel substrates was revealed throughout the vegetation period. Species specificity in the reaction of plants to the presence of gel substrates in the root-inhabited environment was revealed. Lettuce, tomato and cucumber plants were more responsive to the effect of the gel substrate, and radish plants were less responsive. At the same time, more pronounced positive changes in plant growth, quality and productivity were observed in cucumber and lettuce in the variant of gel substrates with macro- and microelements and in tomato plants in both variants of gel substrates. Further research into the mechanisms of the influence of gel substrates on plants, as well as the synthesis of new gel substrates with more pronounced properties to sorb and retain moisture is promising.

## 1. Introduction

The use of hydrogels in agriculture is gradually becoming more in demand. Research is mainly aimed at improving the hydrophysical properties of soils for plants using polymer gels in open ground. As is known, the presence of polymer gels in the soil contributes to the improvement of the provision of plants with moisture and fertilizers contained in hydrogels over a longer period of time, compared to applying fertilizers themselves to the soil. Polymer gels containing macro- and microelements, and compounds with antimicrobial properties necessary for plants can increase competitiveness and stability of seeds germinating in soil or soil substitute, etc. [1,2,3,4,5,6,7,8,9,10,11,12,13,14,15].

Along with this, the positive effect of hydrogels is noted in increasing the resistance of plants to unfavorable environmental conditions, such as climate change, phytopathogens, pesticide load, as well as minimizing the excessive use of fertilizers, pesticides and water in the fields by reducing their losses from root habitats due to gradual release from hydrogels [2,15,16]. All hydrogels are conventionally divided according to the composition and properties of the polymers that form their basis into natural, synthetic and hybrid, and according to the structure of cross-links—into networks with covalent chemical bonds and networks of physical entanglements [17,18]. As is known, the natural hydrogels, having advantages in the biocompatibility, biodegradability and low cost, concede to synthetic polymers in water absorption and retention properties. Therefore, researchers’ attention is drawn to the semi-synthetic polymeric superabsorbents based on natural polymers modified with additives or grafted chains of synthetic polymers [15]. Using them in agriculture might be more effective and promising, especially in dry regions. They can also be used for the slow release of nutrients into the soil, which is important from an environmental point of view. It was reported that the presence of hydrogels in soil provides water and fertilizer retention in soil, reduces evapotranspiration, and improves hydrophysical conditions for plants [19,20,21].

At the same time, hydrogels in greenhouses have not yet found widespread use. Although, according to researchers, the use of gels can be very effective in creating artificial substrates for greenhouses [22,23,24]. Potentially, they could have a positive effect on the plants’ growth and productivity as sources of water and nutrients when introduced into the composition of soil substitutes (based on peat and other organic substrates), as well as due to their coating of seeds.

To date, the issues of optimizing the composition and properties of the root environment of plants in greenhouses conditions remain unresolved. This is a multicomponent task with several variables depending on the specified conditions, as well as the composition and properties of soil substitutes, the production and adaptive potential of the cultivated variety or hybrid of an agricultural crop. It should be noted that the natural mineral, organic and synthetic materials for growing plants widely used in protected grounds crop production do not fully meet the requirements for the root-inhabited environment that provides the optimal conditions for plants’ growth and development throughout ontogenesis [24,25]. This also applies to root-inhabited mediums, represented by nutrient solutions in all known variants of hydroponic and aeroponic methods of growing plants. At the same time, the implementation of these methods requires constant breeding activities to create varieties and hybrids of plants adapted to growing conditions [26].

In this regard, the creation of new materials that (1) have the required properties, (2) have a known stable structure, (3) are capable of transitioning upon contact with water from a light-weight solid form to a gel-like porous form, and (4) are resistant to intensive light culture conditions is a pioneering area of research work with potential practical results, that are very promising for wide industrial use.

To solve this fundamental problem, the efforts of scientists from the Institute of Macromolecular Compounds and the Agrophysical Research Institute carried out research work on the development and testing of new gel substrates based on copolymers of potassium 3-sulfopropyl methacrylate and 2-hydroxyethyl methacrylate, characterized by high water absorption. To date, there are only a few studies in the literature devoted to the potential use of polymers with sulfonic acid groups for agricultural needs [27,28,29]. Thus, the synthesis and study of these hydrogels and gel substrates based on them seems completely new and relevant.

The research hypothesis is as follows: a hydrogel based on copolymers of potassium 3-sulfopropyl methacrylate and 2-hydroxyethyl methacrylate will provide the formation of a substrate necessary for seed germination and plant growth in the early stages of development. The inclusion in its composition of macro- and microelements required for plant growth and development will significantly enhance the beneficial properties of the hydrogel in in terms of the survival and competitiveness of plants using the example of leaf vegetable (lettuce), root vegetable (radish) and fruit vegetable crops (tomato, and cucumber) during their vegetation period.

The purpose of this work is to synthesize the gel substrates based on copolymers of potassium 3-sulfopropyl methacrylate and 2-hydroxyethyl methacrylate and to study their effect on plants using the example of representatives of leaf vegetable (lettuce), root vegetable (radish) and fruit vegetable crops (tomato, and cucumber) to assess the possibility of the gel substrates’ application in greenhouse crop production

The research objectives are as follows:

(1) to synthesize gel substrates based on copolymers of potassium 3-sulfopropyl methacrylate and 2-hydroxyethyl methacrylate with and without the addition of macro- and microelements;
(2) to study under controlled conditions the effect of the created gel substrates on the physiological state of plants (lettuce, radish, cucumber and tomato), namely the content of photosynthetic pigments, the activities of antioxidant enzymes, the intensity of lipid peroxidation, the elemental composition in plant leaves during the vegetative period (lettuce, radish, cucumber and tomato) and generative period of their development (tomato, and cucumber), and plants’ growth and productivity.

## 2. Results and Discussion

### 2.1. Synthesis of Gel Substrates

**Figure 1:**
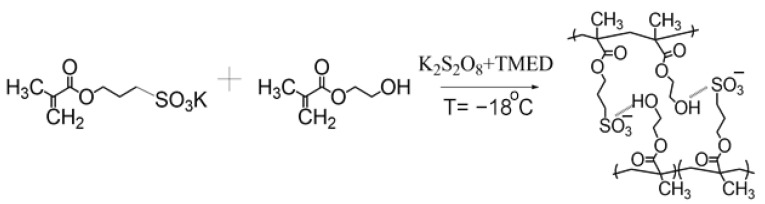
The synthesis scheme of SPMA-co-HEMA.

The synthesis of gels was carried out by copolymerization of 3-sulfopropyl potassium methacrylate (SPMA) and 2-hydroxyethyl methacrylate (HEMA) at a temperature of −18 °C according to the method described in article [29]. The scheme of synthesis is presented in Figure 1. As a result of the use of a comonomer containing a sulfonate group, the gels are characterized by a high swelling degree—205 g/g.

**Figure 2:**
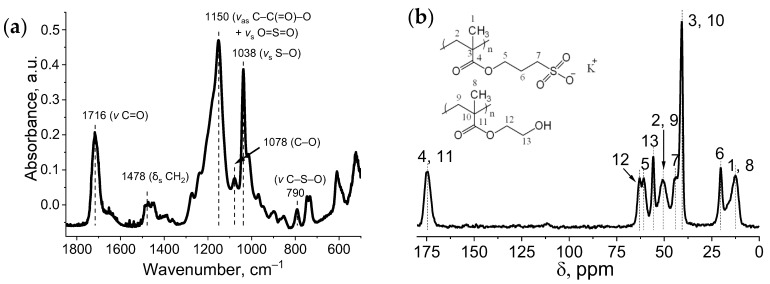
ATR-FTIR (**a**) and CP/MAS ^13^C NMR (**b**) spectra of obtained SPMA-co-HEMA gels.

The structure of the gels was confirmed by ^13^C NMR and ATR-FTIR spectroscopy (Figure 1). The following signals were observed in the ^13^C NMR spectra (Figure 2a): 175 ppm. (carbonyl group), 63 ppm (O−CH_2_) 61 ppm (O−CH_2_), 55 ppm (CH_2_−O) 50 ppm (CH_2_), 44 (CH_2_−SO_3_K), 41 (quaternary carbon), 20 (secondary carbon) and 12 ppm. (methyl group).

The following vibrations were observed in the ATR-FTIR spectra (Figure 2b): 1716 cm^−1^ (*v* C=O), 1478 cm^−1^ (δ_s_ CH_2_), 1150 cm^−1^ (*v*_as_ C−C(=O)−O + vs. O=S=O), 1078 cm^−1^ (C–O) 1038 cm^−1^ (vs. S−O), 790 cm^−1^ (*v* C−S−O), 746 cm^−1^ (ρ CH_2_).

**Figure 3:**
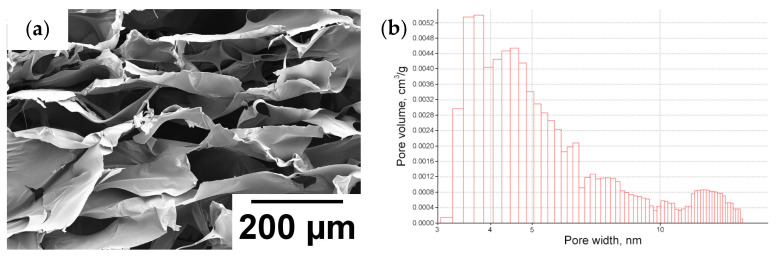
SEM photograph (**a**) and pore size distribution (**b**) of the synthesized gel based on the SPMA-co-HEMA copolymer.

The porous structure of the gel was investigated by SEM and BET analysis. Electron microphotographs of freeze-dried sample are presented in Figure 3a. The obtained gels are high-pore gels. This was confirmed by measurements of porosity by gravimetrical analysis (the porosity is 98%), specific surface area (104 m^2^/g), and pore volume (0.088 cm^3^/g). Moreover, in addition to macropores that were observed on SEM photos, the gel contains a large amount of nano- and mesopores (Figure 3b), as evidenced by the resulting pore size distribution. Such high porosity of gel can positively affect the growth of the plants.

### 2.2. The Influence of the Created Gel Substrates on the Physiological State, Growth and Productivity of Plants

#### 2.2.1. Physiological State of Plants

In a series of vegetation experiments under controlled conditions of an agrobiopolygon, the influence of created gel substrates based on copolymers of potassium 3-sulfopropyl methacrylate and 2-hydroxyethyl methacrylate—SPMA-co-HEMA—with and without enrichment with macro- and microelements on various species of vegetable crops during the periods of their vegetative and generative development was studied. Species specificity was identified in the reaction of the used crops to the presence in the root-inhabited environment of the mentioned gel substrates.

Gel substrates in root habitats have a significant influence on the plants during the vegetative period of their development, judging by the assessed indicators:

- The content of photosynthetic pigments of plants which generally characterize the capacity of the photosynthetic apparatus (Table 1) [30].
- The activity of redox enzymes peroxidase (POX), catalase (CAT), and the intensity of lipid peroxidation (LPO) (Table 2), indicating the direction and effectiveness of the plant antioxidant systems’ functioning [31].
- The content of macro- and microelements in plant leaves at the early stages of their development (Table 3), making it possible to judge the situation with the vital supply of plant micronutrients necessary for growth and development, which are also cofactors of enzymes and constituent elements of pigments and other substances involved in the physiological and metabolic processes of the plant organism [32].

So, the following change in the content of the chlorophylls and carotenoids were identified:

- An increase relative to the control values of the content of chlorophyll a and the total chlorophylls in the leaves of plants in variants of root-inhabited environment with TAS + GG, TAS + mGG for lettuce—by 21–23% and by 10–12%, respectively; for radishes—by 4–5% and by 4–6%; for cucumber—by 7–33% and 5–33%; for tomato—by 18–23% and 21–32% (Table 1). Moreover, the values of these indicators were also in the form of a trend or significantly higher than those in the reference variant when growing plants on root-inhabited environment with TAS + SG in lettuce, radish and tomato plants, and in cucumber plants—only in the variant with root-inhabited environment TAS + GG.
- A significant increase in the chlorophyll b contents values in leaves in variants of root-inhabited environment with TAS + GG, and TAS + mGG relative to the control: in tomato—34 and 62%; in cucumber—significant only in the variant with root-inhabited environment TAS + GG by 33%. For radishes, there are no significant changes; for lettuce, there is a significant or trending decrease in the content of this indicator by 16 and 30% (Table 1). Moreover, the values of this indicator were in the form of a trend or significantly higher than those in the reference variant in the TAS + GG and TAS + mGG variants for radish, in the TAS + GG variant for cucumber, in the TAS + mGG variant for tomato. Lower values were observed in the TAS + mGG variant for lettuce and cucumber, and in the TAS + GG variant for tomato.
- Decreased relative to control values of carotenoid content in plant leaves in variants of root-inhabited environment with TAS + GG or with TAS + mGG in lettuce by 12–26%; in cucumber—only in the TAS + mGG variant by 11% (Table 1). Moreover, compared to the reference variant, which had significantly lower values relative to the control, the variants with gel substrates promote a decrease in its negative effects in lettuce plants, neutralize it for radish, and did not differ in indicators for tomato plants, and had lower values in the TAS + mGG variant by cucumber plants.

The data obtained indicate the activation of the processes of biosynthesis of chlorophyll a in lettuce, tomato and cucumber, chlorophyll b—in tomato and cucumber and, as a result, an increase in total chlorophylls in these plants.

A decrease in the content of chlorophyll b and carotenoids, known for their antioxidant properties [33], in lettuce, of the carotenoids in the variant with the gel substrate TAS + mGG in cucumber plants, as well as the absence of significant changes from the control in the values of the carotenoids in the experimental variants of other phytotest objects apparently indirectly indicates an improvement in the plants state. This is obviously related to a decrease oxidative processes and stabilization of redox reactions in the photosynthetic system. In radish, the direction of these changes is generally similar, but expressed as a weak trend.

This assumption is generally confirmed by the results of assessing changes in the values of parameters characterizing the functioning of plant antioxidant systems. Table 2 shows that, in variants with gel substrates, there is a decrease in the intensity of LPO in the leaves of lettuce (by 12–15%), radish (by 17–32%, more significant in the TAS + GG variant), and tomato (by 9–14%).

The activity of the oxidative enzyme peroxidase (POX) does not differ from the control values in the variants with gel substrates in radish plants, and has a tendency for significantly lower values (by 6–12%) in the variants with TAS + GG and TAS + mGG in the leaves of tomato plants. The activity of the redox enzyme catalase (CAT) in the experimental variants with gel substrates mainly does not differ from the control values, with the exception of the variant with TAS + GG in tomato leaves, where significantly lower values of this indicator were noted (by 10%) relative to the control. At the same time, in the leaves of cucumber plants, a significant increase in LPO values was revealed (by 22–24%) and, as a trend or significantly, in the activity of the POX enzyme (by 11–102%). This may be due to a more pronounced intensification of growth and development processes in cucumber compared to other plants during the period of measuring indicators, and therefore activation of metabolic processes, including redox reactions. It should be noted that there are no differences in the values of indicators between the variants with gel substrates and the reference variant in lettuce and tomato plants, and the more significant manifestation of the described changes in the variants with gel substrates relative to the reference variant in cucumber and radish plants.

Analysis of data on the elemental composition of plant leaves, presented in Table 3, indicates that the presence of hydrogels in the root-inhabited environment significantly affects the content of a number of macro- and microelements during the vegetative period of plant development. In tomato, cucumber and radish, the crude ash content had higher values compared to the control. There was a trend or a significant increase in the content of total nitrogen in the leaves of plants in variants with gel substrates—in cucumber and tomato; phosphorus—in cucumber; potassium—in radish, tomato and in the TAS + GG variant in cucumber; calcium—in tomato (as a trend) and cucumber (reliably in the TAS + mGG variant); magnesium—in lettuce and in cucumber with a maximum in the variant with TAS + mGG, in radish—as a trend in the variant with TAS + GG, in cucumber—in the variants with TAS + GG and TAS + mGG, in tomato—as a trend; manganese—in lettuce with a maximum in the variant with TAS + GG, in radish, in cucumber and in tomato—significantly with a maximum in the variant with TAS + mGG. An increase in copper content was noted only in the variant with TAS + mGG in cucumber. It is interesting to note multidirectional changes in the content of iron in the leaves of the studied crops. It increases as a trend in lettuce leaves, significantly in tomato leaves, but has significantly lower values in radish and cucumber leaves, which may be associated with subsequent redistribution into root vegetables and fruits. The zinc content does not change significantly relative to the control in variants with gel substrates in the leaves of lettuce plants, but increases significantly in tomato, as a trend in cucumber, and has lower values in radish leaves, which is most pronounced in the TAS + GG variant.

#### 2.2.2. Plant Growth and Productivity Indicators of Plant

An increase in the supply of a number of macro- and microelements to plants during the vegetative period of their development, which are, among other things, cofactors of enzymes, and their possible redistribution among plant organs is a favorable factor for the course of metabolic processes, improving the physiological state of plants, which ultimately has a positive effect on plants growth rates.

The latter is confirmed by the corresponding results presented in Table 4 and Table 5. In lettuce plants, cucumber and tomato, there is a significant or trending increase in the values of plant growth indicators at the vegetative stage of development in variants with gel substrates with maximum values in the variant with TAS + mGG, namely the diameter of the rosette in lettuce by 4–14%, the number of leaves in lettuce cucumber and tomato (by 9%, 33–50%, and 2%, respectively), the area of leaves in lettuce, cucumber and tomato (by 10–45%, 96–142% and 48–56%, respectively), dry matter of lettuce and tomato leaves (12% and 6–16%).

In addition to the noted, the cross-sectional area of the stem exceeded the control values by 97–123% in cucumber, by 14–29% in tomato, and the dry matter of roots in cucumber by 47–66%, and in tomato by 20–26%. The noted changes in growth indices in the variants with gel substrates determined the corresponding higher, relative to the control, and reference variant values of wet, dry mass of leaves (by 42–63% and 78–110%), stems (by 34–53% and 92–132%), roots (by 30–75% and 77–264%) in the fruit and vegetable crops of tomato and cucumber, respectively, and in lettuce (by 4–47%).

The revealed pronounced positive effect of the presence of gel substrates around growing seeds and in the root-inhabited environment on the plants’ physiological state, growth indicators during the vegetative period of development of lettuce, radish, cucumber and tomato was reflected in their productivity indicators and production quality.

**Figure 4:**
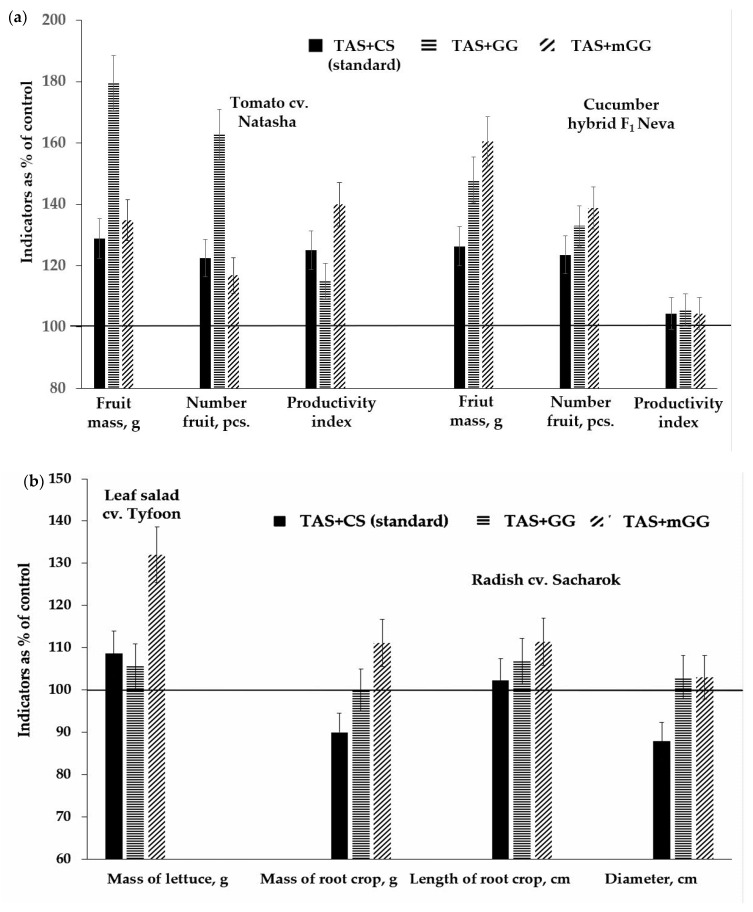
The influence of gel substrates on the growth and productivity of tomato and cucumber plants (**a**) and lettuce and radish (**b**) grown under controlled conditions. Note: TAS—thin-layer analogue of soil; TAS + CS (standard)—thin-layer analogue of soil + clay suspension; TAS + GG—thin-layer analogue of soil + hydrogel; TAS + mGG—thin-layer analogue of soil + hydrogel modified with the addition of macro- and microelements.

It should be noted that very weak changes in the physiological state of the radish practically did not affect the growth rates of the plant aerial part during the vegetative period of development. However, the noted changes in the elemental composition in leaves (Table 3) apparently had a positive effect on the root crops growth, the weight of which tended to be 11% higher in the variant with the gel substrate TAS + mGG relative to that in the control values. At the same time, this indicator’s values in the variant with TAS + GG did not differ from the control (Figure 4b). Judging by the ratio of the mass of root crops to the fresh mass of leaves, called the attraction index [34], the value of this indicator in the form of a tendency only in the variant with the gel substrate TAS + mGG (1.52 ± 0.3) exceeds that in the control (1.35 ± 0.28) by 13%, which also indirectly indicates the redistribution of nutritional elements into root crops from the aerial part of radish plants.

Analysis of productivity data confirms the above assumption regarding the tomato and cucumber. The stimulating effect of gel substrates on plant growth indicators resulted at the generative stage of development of these crops in an increase in the number of fruits and their weight on the plants (Figure 4a). Moreover, these changes relative to the control and reference variants are most pronounced in tomato plants in the variant with the gel substrate TAS + GG, and in cucumber—in the variant TAS + mGG. Higher values of the ratio of fruit weight to fresh leaf weight, significantly or in the form of a trend, in the experimental variants compared to the control indicate the activation of the redistribution of nutrients into fruits from plant leaves. This is more pronounced in tomato plants.

#### 2.2.3. Quality and Safety Indicators of Plant Production

The previously stated assumption about the possible redistribution of a number of microelements from leaves to fruits was confirmed when assessing the elemental composition of the latter. In plant production (lettuce leaves, tomato and cucumber fruits, and radish roots) of the crops used in the experimental variants, including variants with gel substrates, a significant or trending increase in iron content by 7–74% was found (Table 6). The noted changes are most pronounced in cucumber plants in the variant with the gel substrate TAS + mGG, and least pronounced in lettuce. It should be emphasized that, in radish roots and tomato fruits, the iron content values are significantly higher relative to the control values and do not differ from the values in the reference variant.

In the plant production of lettuce, radish, tomato and cucumber, relative to the control, the content of the following elements in the variants with gel substrates increased in the form of a trend or significantly: nitrogen—in cucumber fruits; potassium and phosphorus—in cucumber and tomato; calcium—in cucumber; magnesium—in lettuce and tomato; manganese—in lettuce, tomato and to a lesser extent in cucumber; copper and zinc—in cucumber and tomato (Table 3 and Table 6). An excess in the content of most of the above elements in plant products relative to the reference variant was observed in variants with gel substrates in cucumber plants, in tomato plants only in relation to zinc in the variant with TAS + mGG, and in lettuce plants in relation to magnesium in the same variant of gel substrate.

The content of ascorbic acid in plant products of lettuce, cucumber and tomato was significantly or as a trend higher in the variants with gel substrates compared to the values in the control and reference variants (Table 7). This may be due to the activation of chlorophyll biosynthesis in leaves, an increase in the number, area, mass of leaves, the number of plant stems, and perhaps, as a consequence, there was an increase in the capacity of photosynthesis and the amount of sugars synthesized during photosynthesis (glucose, galactose, etc.), which are precursors of ascorbic acid.

It is interesting to note that the nitrate content in the same variants with gel substrates was significantly lower than the values in the control and reference variants in lettuce and tomato plants and did not differ from the control and less than in the reference variant—in cucumber plants (Table 7). Such opposite changes in the content of ascorbic acid and nitrates are noted in the works of the authors [35,36,37]. It should be noted that in terms of the nitrate content in plant production, the latter in all variants of the experiment complied with the requirements presented in the sanitary and hygienic standards of the Russian Federation and other countries for the nitrate content in the plant production of various vegetable crops.

Thus, the data on the chemical composition of plant products indicate a significant increase in their quality and safety characteristics in variants with gel substrates in a root-inhabited environment.

## 3. Conclusions

This study demonstrated the possibilities of the potential use of gel substrates in plant growing systems under the intensive artificial light, that were created in the process of cryotropic gelation based on the copolymerization of synthesized potassium 3-sulfopropyl methacrylate and 2-hydroxyethyl methacrylate—SPMA-co-HEMA—with and without enrichment with macro- and microelements. Gel substrates have shown themselves to be substances that provide germinating seeds and plants with the necessary moisture and nutrients, and have a regulatory effect on the processes of photosynthetic pigments biosynthesis, and stabilize the antioxidant systems of plants. Using representatives of leaf crops, root crops and fruited vegetables, the positive effect of gel substrates on the physiological state of plants, their growth, productivity and the quality of the resulting plant products was studied and demonstrated.

Species specificity in the reaction of plants to the presence of gel substrates in the root-inhabited environment was revealed. Lettuce, tomato and cucumber plants were more responsive, while radish plants were less responsive. At the same time, the most pronounced changes in growth indicators, plant productivity, elemental composition and other assessed indicators were observed in cucumber and lettuce plants in the variant with gel substrate containing macro- and microelements, and in tomato plants in both variants of gel substrates without and with macro- and microelements. The fact of nutrient redistribution was revealed using the example of iron from leaves to fruits in cucumbers and roots in radishes. The revealed high biological activity of gel substrates emphasizes the prospect of further in-depth studies of the mechanisms of their influence on cultivated plants and their habitat, as well as the synthesis of new gel substrates with more pronounced properties to sorb and retain moisture, gradually providing it and sources of nutrition and energy to growing plants.

## 4. Materials and Methods

### 4.1. Materials

#### 4.1.1. Chemical Materials

3-sulfopropyl methacrylate (SPMA, Sigma-Aldrich, Darmstadt, Germany), *N*,*N*′-methylenebis(acrylamide) (MBA, Sigma-Aldrich, Darmstadt, Germany) *N*,*N*,*N*′,*N*′-tetramethylethylenediamine (TEMED, Sigma-Aldrich, Darmstadt, Germany), 2-hydroxyethyl methacrylate (HEMA) (Sigma-Aldrich, Darmstadt, Germany), potassium persulfate (K_2_S_2_O_8_, JSC Vekton, Saint-Petersburg, Russia), and cyclohexane (JSC Vekton, Saint-Petersburg, Russia) were used. HEMA was purified by dint of molecular sieve. Potassium persulfate was purified by recrystallization from ethanol. Other chemicals were used without preliminary purification. Double-distilled water, with water conductivity 0.1 S/cm was used for carrying out polymerization and research.

#### 4.1.2. Biological Materials

The objects of research were various species of vegetable crops using the example of different varieties or hybrids—leaf salad (*Lactuca sativa* L.) cv. Typhoon, radish (*Raphanus sativus* L.) cv. Sacharok, tomato (*Solanum lycopersicum* L.) cv. Natasha, and cucumber (*Cucumis sativus* L.) hybrid F_1_ Neva. Plant seeds were received from the collection of the Federal Research Center All-Russian Institute of Plant Genetic Resources named after N.I. Vavilov (VIR), Federal Scientific Center for Vegetable Growing, seed companies—joint stock company “Sortsemovoshch” and Group of Companies GAVRISH™. The cultivars of leaf salad, radish, tomato, and cucumber’s test crops were chosen due to their higher adaptability to the controlled conditions of intensive light culture.

### 4.2. Methods

#### 4.2.1. Synthesis of Gels

The synthesis of gels was carried out by cryotropic gelation during copolymerization of potassium 3-sulfopropyl methacrylate (SPMA) with 2-hydroxyethyl methacrylate (HEMA) (SPMA-co-HEMA) according to the method described in [29]. For this, a monomer mixture consisting of SPMA (0.45 g), HEMA (0.2 g), K_2_S_2_O_8_ (0.07 g), TEMED (50 μL) and bidistilled water (5 mL) was kept at −18 °C for 24 h. After that, the gels were washed from unreacted monomers and freeze-dried. The gels were then swollen in a solution containing micro- and macroelements. The composition of macro- and microelements introduced into the hydrogel based on the SPMA-co-HEMA copolymer is presented in [38].

**Figure 5:**
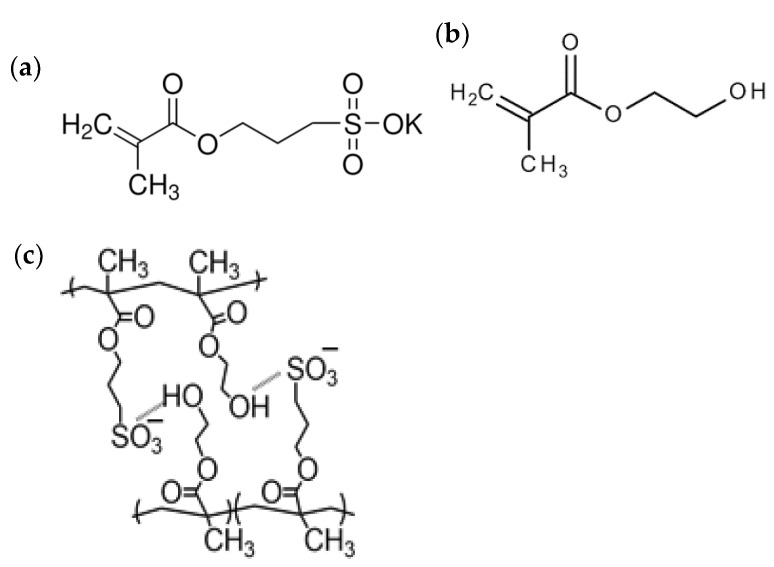
The structure of 3-sulfopropyl potassium methacrylate (**a**), of 2-hydroxyethyl methacrylate (**b**), of co-polymer SPMA-co-HEMA (**c**).

The structure of 3-sulfopropyl potassium methacrylate, of 2-hydroxyethyl methacrylate, and of co-polymer SPMA-co-HEMA is shown in Figure 5.

#### 4.2.2. ATR-FTIR and ^13^C NMR

ATR-FTIR and ^13^C NMR spectroscopy were carried out using freeze-dried samples. The ATR-FTIR spectra were recorded on a spectrometer IR-Affinity-1S (Shimadzu, Kyoto, Japan). Spectroscopy was carried out in the range of wavenumbers 4000–500 cm^−1^. Represented in the present work spectra are an average result of 32 scans.

The solid-state ^13^C NMR spectra were recorded on an AVANCE II-500 WB NMR spectrometer (Bruker, Billerica, MA, USA) operating at the resonance frequency of 125.8 MHz. Polymer samples were packed into zirconium rotors with a diameter of 4 mm; the spectra were registered at a temperature of 20 °C and rotation frequency of 10 or 13 kHz.

#### 4.2.3. Scanning Electron Microscope

SEM images of cryogels were obtained using field emission scanning electron microscopy Zeiss SUPRA 55 VP (Carl Zeiss Industrielle Messtechnik GmbH, Oberkochen, Germany). For SEM, freeze-dried samples were used. Freeze-drying occurred after swelling till the equilibrium state.

#### 4.2.4. Measurement of Specific Surface Area, Pore and Porosity Analysis

The specific surface area was assessed by the nitrogen gas sorption analyzer «NOVA 1200e» (Quantachrome, Boynton Beach, FL, USA). The investigation was held under the Multipoint Brunauer–Emmett–Teller (BET) method. Before measurements, the freeze-dried sample was degassed under nitrogen flow under reduced pressure.

The determination of the total porosity (*P*) of the gels was carried out by gravimetrical analysis. The dried sample was weighed (*m_dry_*), then kept in cyclohexane for an hour to fill the entire pore space, after which the swollen sample was reweighed (*m_swell_*). Total porosity was calculated using the formula:

$$P=\frac{m_{swell}-m_{dry}}{m_{swell}}\cdot 100\%$$

#### 4.2.5. Biological Experiment Design and Conditions

Biological experiments have been conducted at the AFI agrobiopolygon under controlled microclimate conditions (Saint-Petersburg, Russia). There are the in AFI elaborated plant growing light equipment’s (PGLE), where plants can grow under favorable conditions [39]. It used two types of PGLE: for growing the plants with high up to 50 cm and for plants with the formation of a stem height up to 200 cm. Experiment conditions at the AFI agrobiopolygon are presented in Table 8.

##### Experimental Design

The following series of experiments on the use of gel substrates in a system of intensive plant cultivation under controlled conditions were carried out:

1. Evaluation of the influence of the hydrogels introduction to the leaf salad seed on the plants physiological state, biochemical composition, growth, quality of the obtained plant production;
2. Study of the influence of the hydrogels introduction to the radish plants seed on the plants physiological state, biochemical composition, growth, productivity and quality of the obtained plant production;
3. Study of the influence of the hydrogels introduction to the tomato plants seed on the plants physiological state, biochemical composition, growth, productivity and quality of the obtained plant production;
4. Evaluation of the influence of the hydrogels introduction to the cucumber plants seed on the plants physiological state, biochemical composition, growth, productivity and quality of the obtained plant production.

For plant growing, the AFI method of panoponics in the PGLE was employed, where plants roots are located on the thin-layer soil’s analogue. It is a reusable hydrophilic material (polyethylene terephthalate), which is located in the root-inhabited zone of PGLE. The surface of this material serves to accommodate plant seeds and provides plant roots with a nutrient Knop’s solution through the flat slotted capillaries [25,39]. For cucumber, we used the modified Nutrient Knop’s, whose composition is demonstrated in [38].

Options for the formation of root-inhabited environment:

TAS—thin-layer analogue of soil—control;
TAS + CS (standard)—thin-layer analogue of soil + clay [40] suspension (layer 1 mm thick; cation exchange capacity of clay 22.1 mg-eq per 100 g);
TAS + GG dilution 1:500—thin-layer analogue of soil + hydrogel (layer 1 mm thick);
TAS + mGG dilution 1:500—thin-layer analogue of soil + hydrogel modified with the addition of macro- and microelements (layer 1 mm thick).

All experiments were repeated twice.

1. The density of the leaf lettuce cenosis was formed based on 1 m^2^ of usable area of the growing light installation: 40 lettuce plants. The duration of each experiment: 28 days from sowing the seeds to plants aerial part harvesting.
2. Replications per variant by radish—28 plants. The duration of each experiment was 26 days from seed sowing to root vegetable harvesting.
3. Replications per variant by tomato—10 plants. The duration of each experiment was 90 days from seed sowing to fruit harvesting.
4. Replications per variant by cucumber—10 plants. We formed their plants into one stem with a length of 2 m. The duration of each experiment was 58 days from seed sowing to fruit harvesting.

During the growing period, daily monitoring of the condition of the plants was carried out, as well as phenological observations. At the end of the growing period by lettuce, radish plants, in vegetative stadium by cucumber and tomato (on the 25th day and on the 28th day, respectively), the following was measured in plant leaves:

The content of photosynthetic pigments;
The activity of antioxidant enzymes;
The content of macro- and microelements;
The biometric indicators of plant growth: plant leaf area, number of leaves, cross-sectional area of the stem (tomatoes, and cucumbers), length and diameter of root crops (radish), and fresh and dry weight of plant organs (stems, leaves, roots, and % dry substances).

The main indicators of productivity of the cucumber, tomato (the number and weight of fruits on), as well as the quality and safety of plant production were assessed at the end of the growing period.

#### 4.2.6. Plant Analyses

##### Morphology Measurements

Morphology measurements of the leaves area, of the stem cross-sectional area, of the plant raw and dry mass have been made using the methods in [34] and in accordance with the algorithm of sequential actions, described in detail in our previously published article [38].

The productivity index was calculated as the ratio of fruit weight to leaf fresh weight [34].

##### Photosynthetic Pigments Analysis

The chlorophylls a, b and carotenoids were determined with a spectrophotometer PE-3000UV (“Promekolab” LLC, St. Petersburg, Russia) through a measurement of the optical density of the in acetone obtained leaves extracts at wavelengths of 662, 644, and 440.5 nm, respectively [41].

##### Antioxidant System Activity

The antioxidant system activity in the plants roots and aerial parts was assessed by determining the following indicators: the lipid peroxidation’s intensity (LPO), and the peroxidase and catalase enzyme activity. These indicators were determined with methods in [42,43,44]. The algorithm of sequential actions is described in detail in [38].

##### Plant Production’s Quality and Safety Indicators

The plant production’s quality and safety were characterized by determining the values of the following indicators: the content of dry matter, of water-soluble carbohydrates (mono-, disaccharides, and sum of sugars), of ascorbic acid (vitamin C), of raw ash and of macro- and microelements, as well as of nitrates. The evaluation of these indicators values was made with standard and generally accepted methods [45,46,47,48,49]. A description of the analyses carried out is presented in detail in [38].

##### Statistical Analysis

The data were analyzed using one-way analysis of variance (ANOVA) followed by Duncan’s multiple-range test (*p* ≤ 0.05) to determine significant differences between individual means. In the tables and figures presented, the mean ± SE values were calculated using MS Excel 2016 as well as v.12.0 software (StatSoft Inc., Tulsa, OK, USA).

## Figures and Tables

**Table 1:** Effect of the gel substrate on the content of photosynthetic pigments in the leaves of lettuce, radish, cucumber and tomato plants grown under controlled conditions.

Indicators	Leaves			
	Root-Inhabited Environment *			
	TAS (Control)	TAS + SC (Etalon)	TAS + GG	TAS + mGG
Leaf salad cv. Typhoon				
Chlorophyll a, µg 100 g^−1^ FM	70.57 ± 3.53 ^b^	80.24 ± 4.01 ^a^	85.56 ± 4.28 ^a^	87.02 ± 4.35 ^a^
Chlorophyll b, µg 100 g^−1^ FM	22.64 ± 1.13 ^a^	18.59 ± 0.93 ^b^	19.11 ± 0.96 ^b^	15.97 ± 0.80 ^c^
Total chlorophyll, µg 100 g^−1^ FM	93.21 ± 4.66 ^b^	98.83 ± 4.94 ^ab^	104.67 ± 5.23 ^a^	102.99 ± 5.15 ^ab^
Carotenoid, µg 100 g^−1^ FM	25.47 ± 1.27 ^a^	15.98 ± 0.80 ^d^	22.51 ± 1.13 ^b^	18.88 ± 0.94 ^c^
Radish cv. Sacharok				
Chlorophyll a, µg 100 g^−1^ FM	70.83 ± 3.54 ^a^	68.49 ± 3.42 ^a^	73.72 ± 3.69 ^a^	74.39 ± 3.72 ^a^
Chlorophyll b, µg 100 g^−1^ FM	23.07 ± 1.15 ^ab^	22.06 ± 1.10 ^b^	24.17 ± 1.21 ^ab^	24.84 ± 1.24 ^a^
Total chlorophyll, µg 100 g^−1^ FM	93.91 ± 4.70 ^a^	90.55 ± 4.53 ^a^	97.89 ± 4.89 ^a^	99.22 ± 4.96 ^a^
Carotenoid, µg 100 g^−1^ FM	23.24 ± 1.16 ^a^	20.60 ± 1.03 ^b^	23.11 ± 1.16 ^a^	24.25 ± 1.21 ^a^
Cucumber hybrid F_1_ Neva				
Chlorophyll a, µg 100 g^−1^ FM	116.96 ± 5.85 ^b^	125.42 ± 6.27 ^b^	156.01 ± 7.80 ^a^	124.75 ± 6.24 ^b^
Chlorophyll b, µg 100 g^−1^ FM	34.57 ± 1.73 ^b^	36.89 ± 1.84 ^b^	46.03 ± 2.30 ^a^	35.17 ± 1.76 ^b^
Total chlorophyll, µg 100 g^−1^ FM	151.52 ± 7.58 ^b^	162.30 ± 8.12 ^b^	202.05 ± 10.10 ^a^	159.83 ± 7.99 ^b^
Carotenoid, µg 100 g^−1^ FM	40.44 ± 2.02 ^a^	43.94 ± 2.20 ^a^	43.34 ± 2.17 ^a^	35.98 ± 1.80 ^b^
Tomato cv. Natasha				
Chlorophyll a, µg 100 g^−1^ FM	99.00 ± 4.95 ^b^	111.39 ± 5.57 ^a^	116.37 ± 5.82 ^a^	121.76 ± 6.09 ^a^
Chlorophyll b, µg 100 g^−1^ FM	30.02 ± 1.50 ^d^	44.70 ± 2.24 ^b^	40.27 ± 2.01 ^c^	48.61 ± 2.43 ^a^
Total chlorophyll, µg 100 g^−1^ FM	129.02 ± 6.45 ^b^	156.09 ± 7.80 ^a^	156.64 ± 7.83 ^a^	170.37 ± 8.52 ^a^
Carotenoid, µg 100 g^−1^ FM	40.30 ± 2.02 ^a^	43.17 ± 2.16 ^a^	41.69 ± 2.08 ^a^	40.57 ± 2.03 ^a^

Note: * TAS—thin-layer analogue of soil; TAS + CS (standard)—thin-layer analogue of soil + clay suspension; TAS + GG—thin-layer analogue of soil + hydrogel; TAS + mGG—thin-layer analogue of soil + hydrogel modified with the addition of macro- and microelements; FM—leaves’ fresh mass. Different letters (^a–d^) near data in table rows indicate significant differences with their values at *p* ≤ 0.05 (in accordance with Duncan’s multiple range test).

**Table 2:** Effect of the gel substrate on the functioning of antioxidant systems in leaf salad, radish, cucumber and tomato leaves grown under controlled conditions.

Indicators	Leaves			
	Root-Inhabited Environment *			
	TAS (Control)	TAS + SC (Standard)	TAS + GG	TAS + mGG
Leaf salad cv. Typhoon				
CAT, µM H_2_O_2_ mg^−1^ Protein min^−1^	237.39 ± 11.87 ^ab^	237.06 ± 11.85 ^ab^	215.54 ± 10.78 ^b^	250.84 ± 12.54 ^a^
LPO, µM g^−1^	0.0026 ± 0.0001 ^a^	0.0024 ± 0.0001 ^ab^	0.0023 ± 0.0001 ^b^	0.0022 ± 0.0001 ^b^
Radish cv. Sacharok				
POX, U s^−1^ g^−1^	2.08 ± 0.10 ^a^	2.20 ± 0.11 ^a^	2.09 ± 0.10 ^a^	2.27 ± 0.11 ^a^
CAT, µM H_2_O_2_ mg^−1^ Protein min^−1^	325.93 ± 16.30 ^a^	297.61 ± 14.89 ^a^	323.51 ± 16.18 ^a^	318.82 ± 15.94 ^a^
LPO, µM g^−1^	0.0072 ± 0.0004 ^a^	0.0066 ± 0.0003 ^b^	0.0049 ± 0.0002 ^d^	0.0060 ± 0.0003 ^c^
Cucumber hybrid F_1_ Neva				
POX, U s^−1^ g^−1^	8.23 ± 0.41 ^bc^	7.86 ± 0.39 ^c^	9.11 ± 0.46 ^b^	15.87 ± 0.79 ^a^
CAT, µM H_2_O_2_ mg^−1^ Protein min^−1^	443.42 ± 22.17 ^a^	432.53 ± 21.63 ^a^	406.84 ± 20.34 ^a^	404.6 ± 20.23 ^a^
LPO, µM g^−1^	0.0041 ± 0.0002 ^c^	0.0046 ± 0.0003 ^b^	0.0051 ± 0.0003 ^a^	0.0050 ± 0.0003 ^ab^
Tomato cv. Natasha				
POX, U s^−1^ g^−1^	17.02 ± 0.85 ^a^	16.98 ± 0.85 ^a^	15.96 ± 0.80 ^ab^	15.02 ± 0.75 ^b^
CAT, µM H_2_O_2_ mg^−1^ Protein min^−1^	92.0 ± 4.60 ^a^	100.87 ± 5.04 ^a^	83.09 ± 4.15 ^b^	98.35 ± 4.92 ^a^
LPO, µM g^−1^	0.0125 ± 0.0006 ^a^	0.0109 ± 0.0005 ^b^	0.0114 ± 0.0006 ^b^	0.0107 ± 0.0005 ^b^

Note: * TAS—thin-layer analogue of soil; TAS + CS (standard)—thin-layer analogue of soil + clay suspension; TAS + GG dilution 1:500—thin-layer analogue of soil + hydrogel; TAS + mGG dilution 1:500—thin-layer analogue of soil + hydrogel modified with the addition of macro- and microelements. Different letters (^a–d^) near data in table rows indicate significant differences with their values at *p* ≤ 0.05 (in accordance with Duncan’s multiple range test).

**Table 3:** Effect of the gel substrate on the elemental composition in plant leaves during the vegetative period of their development grown under controlled conditions.

Indicators	Leaf Salad cv. Typhoon				Radish cv. Sacharok				Cucumber Hybrid F_1_ Neva				Tomato cv. Natasha			
	Root-Inhabited Environment *															
	TAS (Control)	TAS + SC (Standard)	TAS + GG	TAS + mGG	TAS (Control)	TAS + SC (Standard)	TAS + GG	TAS + mGG	TAS (Control)	TAS + SC (Standard)	TAS + GG	TAS + mGG	TAS (Control)	TAS + SC (Standard)	TAS + GG	TAS + mGG
1	2	3	4	5	6	7	8	9	10	11	12	13	14	15	16	17
Raw ash, % a.d.m.	20.78 ± 1.04 ^a^	21.5 ± 1.08 ^a^	20.60 ± 1.03 ^a^	19.71 ± 0.99 ^a^	24.64 ± 1.23 ^a^	24.53 ± 1.23 ^a^	24.65 ± 1.23 ^a^	24.94 ± 1.25 ^a^	14.46 ± 0.72 ^a^	15.34 ± 0.77 ^a^	15.73 ± 0.79 ^a^	15.60 ± 0.78 ^a^	14.52 ± 0.73 ^b^	16.64 ± 0.83 ^a^	15.75 ± 0.79 ^ab^	15.26 ± 0.76 ^ab^
N, % a.d.m.	4.35 ± 0.22 ^a^	4.51 ± 0.23 ^a^	4.33 ± 0.22 ^a^	4.32 ± 0.22 ^a^	5.25 ± 0.26 ^a^	4.92 ± 0.25 ^a^	5.13 ± 0.26 ^a^	5.17 ± 0.26 ^a^	5.21 ± 0.26 ^a^	5.48 ± 0.27 ^a^	5.48 ± 0.27 ^a^	5.34 ± 0.27 ^a^	4.11 ± 0.21 ^b^	4.80 ± 0.24 ^a^	4.37 ± 0.22 ^ab^	4.50 ± 0.23 ^ab^
P, % a.d.m.	0.81 ± 0.04 ^a^	0.81 ± 0.04 ^a^	0.76 ± 0.04 ^a^	0.79 ± 0.04 ^a^	0.68 ± 0.03 ^ab^	0.74 ± 0.04 ^a^	0.66 ± 0.03 ^b^	0.51 ± 0.03 ^c^	0.65 ± 0.03 ^b^	0.72 ± 0.04 ^a^	0.68 ± 0.03 ^ab^	0.65 ± 0.03 ^b^	0.68 ± 0.03 ^a^	0.73 ± 0.04 ^a^	0.69 ± 0.03 ^a^	0.71 ± 0.04 ^a^
K, % a.d.m.	8.80 ± 0.44 ^b^	9.75 ± 0.49 ^a^	8.70 ± 0.44 ^b^	7.75 ± 0.39 ^c^	4.29 ± 0.21 ^c^	5.20 ± 0.26 ^a^	4.53 ± 0.23 ^bc^	4.79 ± 0.24 ^ab^	2.97 ± 0.15 ^b^	3.32 ± 0.17 ^a^	3.21 ± 0.16 ^ab^	2.95 ± 0.15 ^b^	2.82 ± 0.14 ^b^	3.46 ± 0.17 ^a^	2.90 ± 0.15 ^b^	2.96 ± 0.15 ^b^
Ca, % a.d.m.	1.52 ± 0.08 ^a^	1.49 ± 0.07 ^a^	1.46 ± 0.07 ^a^	1.50 ± 0.08 ^a^	5.88 ± 0.29 ^a^	5.74 ± 0.29 ^a^	5.99 ± 0.30 ^a^	5.88 ± 0.29 ^a^	3.26 ± 0.16 ^b^	3.26 ± 0.16 ^b^	3.41 ± 0.17 ^ab^	3.68 ± 0.18 ^a^	3.10 ± 0.16 ^a^	3.13 ± 0.16 ^a^	3.38 ± 0.17 ^a^	3.25 ± 0.16 ^a^
Mg, % a.d.m.	0.43 ± 0.02 ^c^	0.45 ± 0.02 ^bc^	0.48 ± 0.02 ^ab^	0.52 ± 0.03 ^a^	0.44 ± 0.02 ^ab^	0.43 ± 0.02 ^ab^	0.46 ± 0.02 ^a^	0.41 ± 0.02 ^b^	0.56 ± 0.03 ^b^	0.60 ± 0.03 ^b^	0.67 ± 0.03 ^a^	0.69 ± 0.03 ^a^	0.43 ± 0.02 ^b^	0.54 ± 0.03 ^a^	0.50 ± 0.02 ^b^	0.49 ± 0.02 ^b^
Fe, mg/kg a.d.m.	75.0 ± 3.8 ^a^	78.6 ± 3.9 ^ab^	80.5 ± 4.0 ^ab^	85.6 ± 4.3 ^a^	78.5 ± 3.9 ^a^	63.5 ± 3.2 ^b^	65.9 ± 3.3 ^b^	56.2 ± 2.8 ^c^	70.5 ± 3.5 ^a^	65.6 ± 3.3 ^ab^	62.2 ± 3.1 ^b^	60.8 ± 3.0 ^b^	59.8 ± 3.0 ^b^	72.4 ± 3.6 ^a^	67.4 ± 3.4 ^a^	67.2 ± 3.4 ^a^
Mn, mg/kg a.d.m.	60.2 ± 3.0 ^b^	67.3 ± 3.4 ^a^	70.4 ± 3.5 ^a^	65.1 ± 3.3 ^ab^	135.6 ± 6.8 ^bc^	124.4 ± 6.2 ^c^	146.6 ± 7.3 ^ab^	152.8 ± 7.6 ^a^	239.2 ± 12.0 ^b^	251.8 ± 12.6 ^b^	288.5 ± 14.4 ^a^	301.8 ± 15.1 ^a^	98.0 ± 4.9 ^b^	115.8 ± 5.8 ^a^	114.0 ± 5.7 ^a^	118.7 ± 5.9 ^a^
Cu, mg/kg a.d.m.	5.03 ± 0.25 ^a^	4.86 ± 0.24 ^a^	4.90 ± 0.25 ^a^	4.78 ± 0.24 ^a^	4.47 ± 0.22 ^ab^	4.70 ± 0.24 ^a^	4.18 ± 0.21 ^b^	2.61 ± 0.13 ^c^	8.16 ± 0.41 ^ab^	7.30 ± 0.37 ^c^	7.87 ± 0.39 ^bc^	8.92 ± 0.45 ^a^	4.60 ± 0.23 ^a^	4.83 ± 0.24 ^a^	4.71 ± 0.24 ^a^	4.64 ± 0.23 ^a^
Zn, mg/kg a.d.m.	47.4 ± 2.4 ^a^	47.5 ± 2.4 ^a^	44.1 ± 2.2 ^a^	44.8 ± 2.2 ^a^	68.1 ± 3.4 ^a^	61.4 ± 3.1 ^b^	56.1 ± 2.8 ^b^	58.3 ± 2.9 ^b^	63.5 ± 3.2 ^a^	68.5 ± 3.4 ^a^	66.4 ± 3.3 ^a^	67.3 ± 3.4 ^a^	18.2 ± 0.9 ^b^	22.1 ± 1.1 ^a^	19.7 ± 1.0 ^b^	23.7 ± 1.2 ^a^

Note: * TAS—thin-layer analogue of soil; TAS + CS (standard)—thin-layer analogue of soil + clay suspension; TAS + GG dilution 1:500—thin-layer analogue of soil + hydrogel; TAS + mGG dilution 1:500—thin-layer analogue of soil + hydrogel modified with the addition of macro- and microelements; a.d.m.—absolutely dry matter. Different letters (^a–c^) near data in table rows indicate significant differences with their values at *p* ≤ 0.05 (in accordance with Duncan’s multiple range test).

**Table 4:** Effect of the gel substrate on the growth performance of lettuce and radish plants during the vegetative period of their development grown under controlled conditions.

Root-Inhabited Environment *	Plant						
	Height, cm	Rosette Diameter, cm	Number of Leaves, pcs.	Raw Mass, g	Dry Mass, g	% Dry Matter	Leaves Square, cm^2^
Leaf salad cv. Typhoon							
TAS (control)	14.0 ± 1.2 ^b^	23.0 ± 1.4 ^b^	13.8 ± 1.3 ^a^	46.6 ± 7.0 ^b^	2.40 ± 0.59 ^b^	5.3 ± 0.3 ^ab^	1593.7 ± 378 ^a^
TAS + SC (standard)	15.8 ± 1.2 ^ab^	25.4 ± 1.9 ^ab^	14.0 ± 1.5 ^a^	50.6 ± 8.2 ^ab^	2.42 ± 0.76 ^ab^	4.8 ± 0.2 ^b^	1728.6 ± 518 ^a^
TAS + GG	16.1 ± 1.2 ^a^	23.9 ± 1.4 ^ab^	13.0 ± 1.3 ^a^	49.2 ± 9.9 ^ab^	2.50 ± 0.56 ^ab^	5.1 ± 0.3 ^b^	1840.4 ± 374 ^a^
TAS + mGG	15.9 ± 0.9 ^ab^	26.2 ± 1.9 ^a^	15.0 ± 1.0 ^a^	61.5 ± 1.1 ^a^	3.53 ± 0.39 ^a^	5.7 ± 0.3 ^a^	2215.9 ± 245 ^a^
Radish cv. Sacharok							
TAS (control)	24.4 ± 0.9 ^a^	23.1 ± 0.9 ^ab^	5.8 ± 0.6 ^a^	22.2 ± 3.4 ^a^	1.64 ± 0.26 ^ab^	7.4 ± 0.2 ^b^	828.7 ± 132 ^a^
TAS + SC (standard)	23.5 ± 1.1 ^a^	24.7 ± 1.5 ^a^	5.7 ± 0.4 ^a^	23.7 ± 5.0 ^a^	1.66 ± 0.35 ^ab^	7.0 ± 0.3 ^b^	949.8 ± 200 ^a^
TAS + GG	22.9 ± 1.8 ^a^	22.2 ± 1.4 ^ab^	5.9 ± 0.2 ^a^	22.4 ± 3.3 ^a^	1.78 ± 0.26 ^a^	7.9 ± 0.3 ^a^	880.1 ± 149 ^a^
TAS + mGG	22.6 ± 1.0 ^a^	21.9 ± 1.1 ^b^	5.5 ± 0.3 ^a^	19.8 ± 2.4 ^a^	1.27 ± 0.15 ^b^	6.4 ± 0.2 ^c^	741.7 ± 96 ^a^

Note: * TAS—thin-layer analogue of soil; TAS + CS (standard)—thin-layer analogue of soil + clay suspension; TAS + GG dilution 1:500—thin-layer analogue of soil + hydrogel; TAS + mGG dilution 1:500—thin-layer analogue of soil + hydrogel modified with the addition of macro- and microelements. Different letters (^a–c^) near data in table columns indicate significant differences with their values at *p* ≤ 0.05 (in accordance with Duncan’s multiple range test).

**Table 5:** Effect of the gel substrate on the growth performance of cucumber and tomato plants during the vegetative period grown under controlled conditions.

Root-Inhabited Environment *	Height, cm	Leaves/Plant					Stems/Plant				Roots/Plant			
		Number of Leaves, pcs	Leaves Square, cm^2^	Raw Mass, g	Dry Mass, g	% Dry Matter	Cross-Sectional Area, cm^2^	Raw Mass, g	Dry Mass, g	% Dry Matter	Height, cm	Raw Mass, g	Dry Mass, g	% Dry Matter
Cucumber Hybrid F_1_ Neva														
TAS (control)	9.5 ± 1.5 ^c^	3.0 ± 0.0 ^c^	607 ± 74 ^d^	10.8 ± 1.3 ^c^	1.64 ± 0.07 ^c^	15.1 ± 0.3 ^a^	33.0 ± 0.9 ^d^	6.0 ± 0.7 ^c^	0.31 ± 0.04 ^c^	5.2 ± 0.2 ^a^	33.3 ± 0.9 ^a^	7.9 ± 1.5 ^c^	0.25 ± 0.05 ^c^	3.2 ± 0.2 ^c^
TAS + SC (standard)	12.2 ± 1.2 ^b^	3.6 ± 0.4 ^bc^	925 ± 98 ^c^	15.3 ± 3.9 ^bc^	2.24 ± 0.58 ^bc^	14.6 ± 0.2 ^a^	48.1 ± 2.7 ^c^	8.5 ± 2.7 ^bc^	0.44 ± 0.13 ^bc^	5.1 ± 0.1 ^a^	37.0 ± 5.3 ^a^	10.7 ± 3.0 ^bc^	0.52 ± 0.03 ^bc^	4.9 ± 0.3 ^ab^
TAS + GG	14.2 ± 1.6 ^ab^	4.0 ± 0.2 ^b^	1193 ± 139 ^b^	19.8 ± 2.2 ^ab^	2.90 ± 0.57 ^ab^	14.7 ± 0.3 ^a^	65.0 ± 0.8 ^b^	11.5 ± 1.9 ^ab^	0.60 ± 0.15 ^ab^	5.2 ± 0.2 ^a^	35.0 ± 2.7 ^a^	14.0 ± 2.7 ^ab^	0.66 ± 0.21 ^ab*^	4.7 ± 0.3 ^b^
TAS + mGG	15.3 ± 1.1 ^a^	4.6 ± 0.3 ^a^	1469 ± 193 ^a^	22.7 ± 2.7 ^a^	3.38 ± 0.41 ^a^	14.9 ± 0.2 ^a^	73.6 ± 3.4 ^a^	13.9 ± 2.3 ^a^	0.73 ± 0.11 ^a^	5.3 ± 0.2 ^a^	35.0 ± 2.4 ^a^	17.3 ± 3.4 ^a^	0.91 ± 0.11 ^a*^	5.3 ± 0.2 ^a^
Tomato cv. Natasha														
TAS (control)	16.0 ± 0.9 ^a^	8.4 ± 0.4 ^a^	818 ± 73 ^b^	25.0 ± 2.2 ^c^	2.7 ± 0.2 ^b^	10.6 ± 0.4 ^b^	30.6 ± 1.8 ^c^	9.6 ± 1.5 ^b^	1.0 ± 0.2 ^b^	10.2 ± 0.2 ^b^	30.8 ± 1.7 ^a^	12.7 ± 0.9 ^b^	0.8 ± 0.1 ^c^	6.5 ± 0.2 ^b^
TAS + SC (standard)	16.8 ± 0.8 ^a^	8.4 ± 0.4 ^a^	1141 ± 95 ^a^	30.5 ± 2.5 ^b^	2.7 ± 0.2 ^b^	8.7 ± 0.5 ^c^	29.8 ± 1.7 ^c^	9.8 ± 0.6 ^b^	0.9 ± 0.1 ^b^	8.7 ± 0.4 ^c^	34.2 ± 3.2 ^a^	13.3 ± 1.8 ^ab^	1.0 ± 0.1 ^bc^	7.6 ± 0.3 ^a^
TAS + GG	16.8 ± 0.7 ^a^	8.4 ± 0.4 ^a^	1214 ± 131 ^a^	35.6 ± 3.9 ^a^	4.4 ± 0.5 ^a^	12.3 ± 0.4 ^a^	34.8 ± 2.1 ^b^	12.9 ± 1.0 ^a^	1.4 ± 0.2 ^a^	11.1 ± 0.3 ^a^	35.8 ± 4.6 ^a^	16.5 ± 1.4 ^a^	1.4 ± 0.2 ^a^	8.2 ± 0.3 ^a^
TAS + mGG	17.4 ± 1.1 ^a^	8.6 ± 0.4 ^a^	1276 ± 128 ^a^	39.0 ± 3.9 ^a^	4.4 ± 0.4 ^a^	11.2 ± 0.2 ^b^	39.4 ± 2.2 ^a^	14.7 ± 1.6 ^a^	1.5 ± 0.2 ^a^	10.4 ± 0.2 ^b^	30.6 ± 1.6 ^a^	15.9 ± 2.5 ^ab^	1.2 ± 0.2 ^ab^	7.8 ± 0.4 ^a^

Note: * TAS—thin-layer analogue of soil; TAS + CS (standard)—thin-layer analogue of soil + clay suspension; TAS + GG dilution 1:500—thin-layer analogue of soil + hydrogel; TAS + mGG dilution 1:500—thin-layer analogue of soil + hydrogel modified with the addition of macro- and microelements. Different letters (^a–d^) near data in table columns indicate significant differences with their values at *p* ≤ 0.05 (in accordance with Duncan’s multiple range test).

**Table 6:** Effect of the gel substrate on the elemental composition in radish roots and tomato, cucumber fruits grown under controlled conditions.

Indicators	Radish cv. Sacharok				Cucumber Hybrid F_1_ Neva				Tomato cv. Natasha			
	Root-Inhabited Environment *											
	TAS (Control)	TAS + SC (Standard)	TAS + GG	TAS + mGG	TAS (Control)	TAS + SC (Standard)	TAS + GG	TAS + mGG	TAS (Control)	TAS + SC (Standard)	TAS + GG	TAS + mGG
1	2	3	4	5	6	7	8	19	10	11	12	13
Raw ash, % a.d.m.	16.24 ± 0.81 ^a^	15.85 ± 0.79 ^a^	16.14 ± 0.81 ^a^	15.82 ± 0.79 ^a^	11.83 ± 0.59 ^a^	12.15 ± 0.61 ^a^	12.10 ± 0.61 ^a^	12.22 ± 0.61 ^a^	9.84 ± 0.49 ^b^	9.93 ± 0.50 ^ab^	10.86 ± 0.54 ^a^	9.89 ± 0.49 ^ab^
N, % a.d.m.	2.83 ± 0.14 ^a^	3.06 ± 0.15 ^a^	2.97 ± 0.15 ^a^	2.97 ± 0.15 ^a^	3.87 ± 0.19 ^b^	3.85 ± 0.19 ^b^	4.19 ± 0.21 ^ab^	4.32 ± 0.22 ^a^	2.60 ± 0.13 ^a^	2.81 ± 0.14 ^a^	2.67 ± 0.13 ^a^	2.80 ± 0.14 ^a^
P, % a.d.m.	0.76 ± 0.04 ^a^	0.79 ± 0.04 ^a^	0.73 ± 0.04 ^a^	0.76 ± 0.04 ^a^	0.94 ± 0.05 ^b^	0.97 ± 0.05 ^b^	1.06 ± 0.05 ^a^	0.99 ± 0.05 ^b^	0.61 ± 0.03 ^c^	0.72 ± 0.03 ^a^	0.60 ± 0.04 ^bc^	0.68 ± 0.03 ^ab^
K, % a.d.m.	7.42 ± 0.37 ^a^	7.25 ± 0.36 ^a^	7.41 ± 0.37 ^a^	7.41 ± 0.37 ^a^	5.45 ± 0.27 ^a^	5.43 ± 0.27 ^a^	5.68 ± 0.28 ^a^	5.82 ± 0.29 ^a^	4.16 ± 0.21 ^b^	5.02 ± 0.25 ^a^	4.34 ± 0.22 ^b^	4.18 ± 0.21 ^b^
Ca, % a.d.m.	0.60 ± 0.03 ^a^	0.60 ± 0.03 ^a^	0.60 ± 0.03 ^a^	0.58 ± 0.03 ^a^	0.52 ± 0.03 ^b^	0.52 ± 0.03 ^b^	0.55 ± 0.03 ^b^	0.70 ± 0.04 ^a^	0.18 ± 0.01 ^a^	0.19 ± 0.01 ^a^	0.18 ± 0.01 ^a^	0.18 ± 0.01 ^a^
Mg, % a.d.m.	0.17 ± 0.01 ^a^	0.17 ± 0.01 ^a^	0.18 ± 0.01 ^a^	0.18 ± 0.01 ^a^	0.26 ± 0.01 ^a^	0.26 ± 0.01 ^a^	0.26 ± 0.01 ^a^	0.28 ± 0.01 ^a^	0.14 ± 0.01 ^b^	0.16 ± 0.01 ^a^	0.17 ± 0.01 ^a^	0.17 ± 0.01 ^a^
Fe, mg/kg a.d.m.	17.9 ± 0.9 ^d^	28.2 ± 1.4 ^a^	20.5 ± 1.0 ^c^	23.1 ± 1.2 ^b^	31.8 ± 1.6 ^c^	35.2 ± 1.8 ^c^	43.5 ± 2.2 ^b^	55.2 ± 2.8 ^a^	30.3 ± 1.5 ^b^	37.0 ± 1.8 ^a^	35.5 ± 1.8 ^a^	36.6 ± 1.8 ^a^
Mn, mg/kg a.d.m.	17.9 ± 0.9 ^a^	17.0 ± 0.8 ^a^	16.4 ± 0.8 ^a^	17.3 ± 0.9 ^a^	36.4 ± 1.8 ^a^	36.0 ± 1.8 ^a^	37.9 ± 1.9 ^a^	38.4 ± 1.9 ^a^	11.8 ± 0.6 ^b^	13.9 ± 0.7 ^a^	14.3 ± 0.7 ^a^	14.9 ± 0.7 ^a^
Cu, mg/kg a.d.m.	2.59 ± 0.13 ^a^	2.54 ± 0.13 ^a^	2.55 ± 0.13 ^a^	2.57 ± 0.13 ^a^	10.30 ± 0.52 ^c^	9.90 ± 0.50 ^c^	15.10 ± 0.76 ^b^	19.20 ± 0.96 ^a^	10.02 ± 0.50 ^c^	12.80 ± 0.64 ^a^	11.50 ± 0.58 ^b^	11.70 ± 0.59 ^b^
Zn, mg/kg a.d.m.	47.8 ± 2.4 ^ab^	48.0 ± 2.4 ^ab^	44.1 ± 2.2 ^b^	49.0 ± 2.4 ^a^	28.3 ± 1.4 ^c^	27.2 ± 1.4 ^c^	44.5 ± 2.2 ^b^	51.2 ± 2.6 ^a^	16.2 ± 0.8 ^c^	18.3 ± 0.9 ^b^	17.6 ± 0.9 ^bc^	21.4 ± 1.1 ^a^

Note: * TAS—thin-layer analogue of soil; TAS + CS (standard)—thin-layer analogue of soil + clay suspension; TAS + GG dilution 1:500—thin-layer analogue of soil + hydrogel; TAS + mGG dilution 1:500—thin-layer analogue of soil + hydrogel modified with the addition of macro- and microelements; a.d.m.—absolutely dry matter. Different letters (^a–c^) near data in table rows indicate significant differences with their values at *p* = 0.05 (in accordance with Duncan’s multiple range test).

**Table 7:** Effect of gel substrate on the biochemical composition of plant production formed during cultivation under controlled conditions.

Indicators	Leaf Salad cv. Typhoon				Radish cv. Sacharok				Cucumber Hybrid F_1_ Neva				Tomato cv. Natasha			
	Root-Inhabited Environment *															
	TAS (Control)	TAS + SC (Standard)	TAS + GG	TAS + mGG	TAS (Control)	TAS + SC (Standard)	TAS + GG	TAS + mGG	TAS (Control)	TAS + SC (Standard)	TAS + GG	TAS + mGG	TAS (Control)	TAS + SC (Standard)	TAS + GG	TAS + mGG
Humidity,%	94.56 ± 4.73 ^a^	93.44 ± 4.67 ^a^	95.10 ± 4.76 ^a^	94.87 ± 4.74 ^a^	94.80 ± 4.74 ^a^	94.40 ± 4.72 ^a^	94.40 ± 4.72 ^a^	94.60 ± 4.73 ^a^	96.80 ± 4.84 ^a^	96.60 ± 4.83 ^a^	96.80 ± 4.84 ^a^	96.80 ± 4.84 ^a^	94.89 ± 4.74 ^a^	94.84 ± 4.74 ^a^	94.82 ± 4.74 ^a^	94.64 ± 4.73 ^a^
% dry matter	5.44 ± 0.27 ^b^	6.56 ± 0.33 ^a^	4.90 ± 0.25 ^b^	5.13 ± 0.26 ^b^	5.20 ± 0.26 ^a^	5.60 ± 0.28 ^a^	5.60 ± 0.28 ^a^	5.40 ± 0.27 ^a^	3.20 ± 0.16 ^a^	3.40 ± 0.17 ^a^	3.20 ± 0.16 ^a^	3.40 ± 0.17 ^a^	5.11 ± 0.26 ^a^	5.16 ± 0.26 ^a^	5.18 ± 0.26 ^a^	5.36 ± 0.27 ^a^
Vitamin C, mg/100 g FM	15.84 ± 0.79 ^b^	12.10 ± 0.61 ^c^	17.16 ± 0.86 ^ab^	18.25 ± 0.91 ^a^	21.30 ± 1.07 ^a^	18.70 ± 0.94 ^b^	22.00 ± 1.10 ^a^	22.00 ± 1.12 ^a^	4.62 ± 0.23 ^b^	5.06 ± 0.25 ^b^	9.02 ± 0.45 ^a^	9.41 ± 0.47 ^a^	15.20 ± 0.76 ^b^	16.50 ± 0.83 ^b^	19.80 ± 0.99 ^a^	20.90 ± 1.05 ^a^
Nitrates, mg/kg FM	1200.4 ± 60.02 ^b^	1807.0 ± 90.35 ^a^	664.9 ± 33.25 ^c^	715.2 ± 35.76 ^c^	1192.0 ± 71.0 ^a^	1186.0 ± 76.6 ^a^	1206.0 ± 62.4 ^a^	1171.0 ± 58.6 ^a^	277.0 ± 13.85 ^b^	340.0 ± 17.00 ^a^	283.0 ± 14.15 ^b^	297.0 ± 14.85 ^b^	150.0 ± 7.50 ^a^	105.0 ± 5.25 ^b^	71.3 ± 3.57 ^c^	66.6 ± 3.33 ^c^

Note: * TAS—thin-layer analogue of soil; TAS + CS (standard)—thin-layer analogue of soil + clay suspension; TAS + GG dilution 1:500—thin-layer analogue of soil + hydrogel; TAS + mGG dilution 1:500—thin-layer analogue of soil + hydrogel modified with the addition of macro- and microelements; FM—fresh mass of leaves. Different letters (^a–c^) near data in table rows indicate significant differences with their values at *p* = 0.05 (in accordance with Duncan’s multiple range test).

**Table 8:** Experiments conditions at the AFI agrobiopolygon.

Plant	Growing Equipment	Method of Plant Cultivation	Nutrient Solution	Method of Treatment with Test Substance	Condition					
					Air Temperature: Day/Night	Air Humidity	Light Source/Duration of the Light Period	Light Intensity	pH of Root Inhabitat Zone, Relative Units	EC, mS cm^−1^
Leaf salad	Plant growing light equipment for plants with High up to 50 cm	Panoponic [25]	Knop	Introduction the seeds	+20–+22 °C/ +16–+18 °C	60–70%	High-pressure sodium lamps (DNaZ-400, “Reflax” LLC, Moscow, Russia)/14 h per day	80–90 W/m^2^ in the PAR	5.8–6.0	1.5
Radish					+20–+22 °C/ +16–+18 °C	60–70%				
Tomato					+22–+24 °C/ +18–+20 °C	60–70%	High-pressure sodium lamps (DNaZ-400, “Reflax” LLC, Moscow, Russia)/16 h per day			
Cucum-ber	Plant growing light equipment for plants with High up to 200 cm		modified Knop [38]		+22–+24 °C/ +18–+20 °C	75–80%	High-pressure sodium lamps (DNaZ-400, “Reflax” LLC, Moscow, Russia)/14 h per day		Nutrint solution 6.0–6.2	Nutrint solution 1.6

## Data Availability

The data presented in this study are available upon request from the corresponding author.
